# The Influence of Winter Swimming on Oxidative Stress Indicators in the Blood of Healthy Males

**DOI:** 10.3390/metabo13020143

**Published:** 2023-01-17

**Authors:** Roland Wesołowski, Celestyna Mila-Kierzenkowska, Marta Pawłowska, Karolina Szewczyk-Golec, Łukasz Saletnik, Paweł Sutkowy, Alina Woźniak

**Affiliations:** 1 Department of Medical Biology and Biochemistry, Faculty of Medicine, Ludwik Rydygier Collegium Medicum in Bydgoszcz, Nicolaus Copernicus University in Toruń, 87-100 Toruń, Poland; 2 Department of Vascular and Internal Diseases, Faculty of Health Sciences, Ludwik Rydygier Collegium Medicum in Bydgoszcz, Nicolaus Copernicus University in Toruń, 87-100 Toruń, Poland

**Keywords:** antioxidant enzymes, cold water immersion, lipid peroxidation, oxidative stress, winter swimming

## Abstract

Baths in cold water are a popular physical activity performed to improve health. This study aimed to determine whether repeated cold-water exposure leads to the up-regulation of antioxidant defenses and whether or not this leads to a reduction in basal and/or acute pulses of oxidative distress in humans. The study group consisted of 28 healthy male members of the WS club (average age 39.3 ± 6.1 years). The study sessions occurred at the beginning and the end of the WS season. During the WS season, the participants took 3-min cold-water baths in a cold lake once a week. Blood samples were collected three times during each session: before the bath, 30 min after the bath, and 24 h after the bath. The activity of selected antioxidant enzymes, including superoxide dismutase (SOD), catalase, and glutathione peroxidase (GPx), as well as the concentration of lipid peroxidation (LPO) products, including thiobarbituric acid-reactive substances (TBARS) and conjugated dienes (CD), were determined in erythrocytes. The concentration of TBARS, CD, retinol, and α-tocopherol were determined in the blood plasma, whereas the level of other LPO products, including 4-hydroxynonenal and 8-iso-prostaglandin F2α, were determined in the blood serum. The repeated cold exposure up-regulated most antioxidant defenses, and this led to an attenuation of most indicators of oxidative stress at the baseline and acute pulses in response to cold exposure. In conclusion, due to regular cold exposure, the antioxidant barrier of winter swimmers was stimulated. Thus, short cold-bath sessions seem to be an effective intervention, inducing promoting positive adaptive changes such as the increased antioxidant capacity of the organism.

## 1. Introduction

Oxygen is an element crucial for the functioning of aerobic organisms. However, all organisms that breathe oxygen are also exposed to the harmful consequences resulting from its transformations. Excess O_2_ can lead to the formation of reactive oxygen species (ROS), which, apart from their physiological functions, also have harmful effects [1]. In aerobic organisms, small amounts of ROS and nitrogen free radicals under physiological conditions are constantly generated under the influence of external and internal stimuli [2,3]. Oxygen homeostasis is, therefore, one of the critical conditions that must be maintained for aerobic organisms to properly function [1,4].

Oxidative stress occurs due to the increased formation of free oxygen radicals and/or weakened functioning of the antioxidant barrier [3]. An uncontrolled release of free radicals and their derivatives by damaging nucleic acids, enzymes, and biological membranes leads to the development of pathological conditions [5]. Oxygen free radicals are responsible for lipid peroxidation (LPO) [3]. This process consists of the free-radical oxidation of unsaturated fatty acids or other lipids, resulting in the formation of peroxides of these compounds [6]. In LPO, oxygen free radicals remove electrons from lipids and produce reactive intermediates that can undergo further reactions [7,8]. LPO products can cause DNA damage and directly inhibit numerous proteins [2]. Extensive peroxidation in cell membranes causes changes in their fluidity, increased permeability, reduction of membrane potential, and ultimately the rupture of cell membranes [7]. The most frequently determined markers of lipid peroxidation include thiobarbituric acid-reactive substances (TBARS) [2]. Among the TBARS, malondialdehyde (MDA), the final product of lipid degradation caused by oxidative stress, is the most important compound [2]. Among lipid peroxidation products, 4-hydroxynonenal (4-HNE, 4-hydroxy-2,3-trans-nonenal) and isoprostanes also perform an important role. 8-iso-prostaglandin F2α (F2-isoprostane; 8-isoprostane; 8-iso-PGF2α; 8-isoP) is one of the better-known isoprostanes. Isoprostanes are generated via the mechanism of free radical formation in membrane lipids. Therefore, their formation may affect the fluidity and integrity of cell membranes [9,10,11].

The antioxidant system includes antioxidant enzymes and non-enzymatic antioxidants. Superoxide dismutase SOD is an enzyme that catalyzes the dismutation reaction of the superoxide anion radical to O_2_ and the less reactive H_2_O_2_ [2,12]. The resulting hydrogen peroxide is then degraded by catalase (CAT) or glutathione peroxidase (GPx) [13]. In addition to enzymatic defense, small-molecular antioxidants, including vitamins, play an important protective role against the adverse effects of ROS.

Cold is often used in medicine to reduce inflammation [14]. As a result of winter swimming, physiological changes occur immediately, while repeated exposure to cold develops adaptive mechanisms that also affect health [15]. The type of cold adaptation depends on the intensity of cold stress and individual factors such as body fat percentage, general physical activity, and diet [15]. There are few studies describing the effect of single baths in cold water on the oxidant–antioxidant balance in the human organism. Exposure to cold water induces a significant stress response similar to acute exercise, with increases in cortisol, epinephrine, and norepinephrine [16]. Acute exercise and cold exposure are known to increase levels of peroxisome proliferator-activated receptor γ coactivator-1 (PGC-1α) in muscle and adipose tissue [17,18], and this is known to lead to an increase in antioxidant defense [19]. However, there is no data on the impact of regular winter swimming throughout the autumn and winter seasons. Therefore, as part of this study, it was decided to determine the tested parameters in the same people both at the beginning (October) and at the end (April) of the winter swimming season. This study aimed to determine whether repeated cold-water exposure led to the up-regulation of antioxidant defenses and whether or not this led to a reduction in basal and/or acute pulses of oxidative distress in humans.

## 2. Materials and Methods

### 2.1. Participants

This study covered a group of 28 healthy male volunteers (average age 39.3 ± 6.1 years) who were members of a winter swimming club. Only people who regularly (at least once a week) bathed in cold and icy water for at least two previous seasons took part in the study. Before and during the study, the participants did not change their eating habits or physical activity. Eligibility criteria are presented in Table 1.

The measurement of body components was carried out using the bioelectrical impedance analyzer—Tanita BC 418 MA (Tanita Corporation, Tokyo, Japan). The body composition analysis of the study participants is presented in Table 2.

The study was approved by the Bioethical Committee operating at Ludwik Rydygier Collegium Medicum in Bydgoszcz of Nicolaus Copernicus University in Toruń, Poland (approval number: KB 514/2014). Subjects participating in the experiment became familiar with the assumptions of the experiment. The study participants were also informed about the possibility of resigning from participation in the experiment at any stage without giving a reason, and they gave their written consent to participate in the study.

### 2.2. Study Design

**Figure 1:**
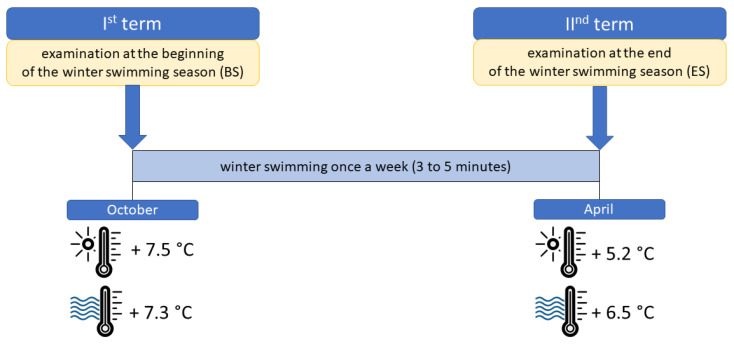
The schedule of material collection and the air and water temperature on the research days.

The winter swimming season usually starts in autumn and lasts until spring, so the study was divided into two stages. The first stage took place at the beginning of the season (BS), i.e., in October, while the second stage of the research took place at the end of the winter swimming season (ES), i.e., in April (Figure 1). During the season (between the first and second dates of blood collection), the participants took cold baths once a week.

**Figure 2:**
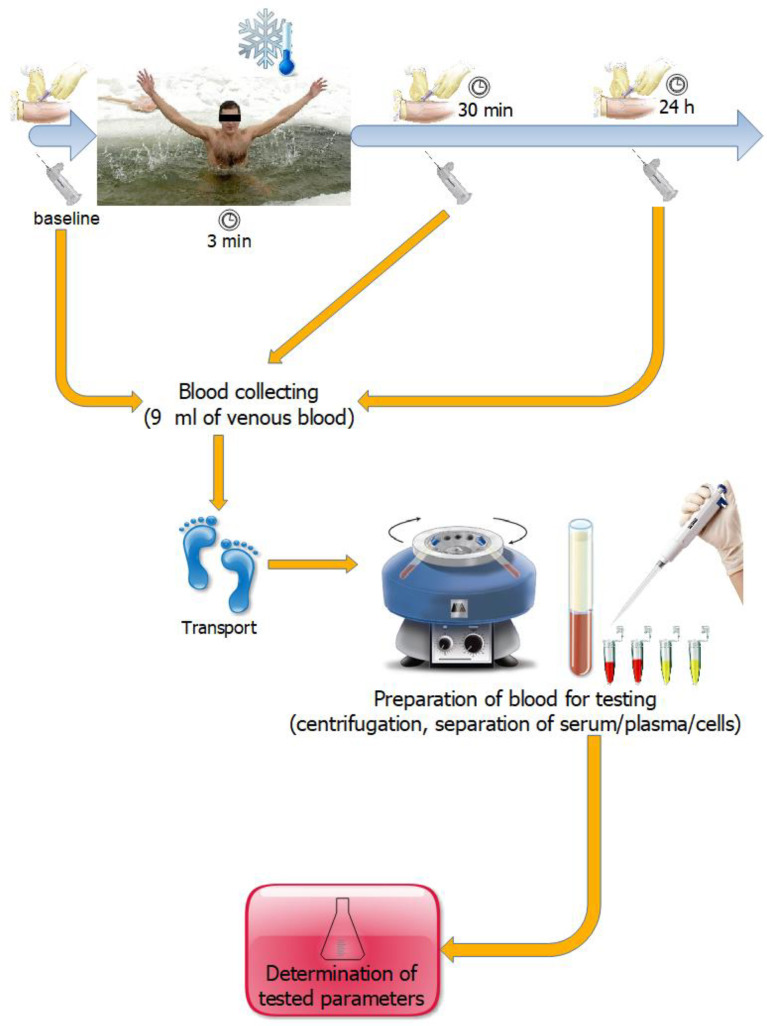
Collecting material for research in each of the two stages of the experiment.

In both stages of the study, the bath consisted of a 3-min immersion in a cold lake to the depth determined by the nipple line. The water temperature was 7.3 °C at BS and 6.5 °C at ES. During the bath, the study participants were dressed in swimwear (slips), a winter hat, and gloves, as is customary during winter swimming. In order to protect their feet from injury, swimmers wore special water shoes. Before entering the water, the study participants performed a short (5-min) moderate-intensity warm-up. The experiment conditions did not differ between the two stages of the study. The study participants were dressed as they were on the first day of the experiment, performed the same warm-up, and immersed themselves in the water to the same depth. Venous blood collection was carried out at a medical point, a part of the bathing infrastructure, located by the beach. Blood for testing was collected by authorized and qualified medical personnel. At each of the two study dates, blood samples were collected three times (Figure 2):

- At the beginning of the swimming season (BS) (in October):
  ∘ Before swimming in cold water (baseline) (BS-0);
  ∘ 30 min after a bath in cold water (BS-30);
  ∘ 24 h after a cold-water bath (BS-24).

- At the end of the swimming season (ES) (in April):
  ∘ Before swimming in cold water (baseline) (ES-0);
  ∘ 30 min after a bath in cold water (ES-30);
  ∘ 24 h after a cold-water bath (ES-24).

The material collected for biochemical tests included venous blood specimens collected in a vacuum system from the basilic vein. For serum collection, blood samples were collected into tubes containing a clotting activator (SiO_2_) and a separating gel. Determination of parameters in erythrocytes and blood plasma was performed using blood samples collected into test tubes containing the anticoagulant dipotassium edetate (K_2_EDTA).

TBARS concentration and SOD, CAT, and GPx activities were determined via spectrophotometric methods using a Cary 60 UV-Vis spectrophotometer (Agilent Technologies, Santa Clara, CA, USA). Concentrations of vitamins A and E were determined using high-performance liquid chromatography (HPLC) with the ProStar System kit with a fluorescence detector (Varian, Palo Alto, CA, USA). In contrast, the concentrations of 4-HNE and 8-iso-PGF2α were determined using commercial diagnostic kits based on the immunoenzymatic method (ELISA) using a SPECTROstar Nano plate spectrophotometer (BMG LABTECH, Ortenberg, Germany). All tested parameters were analyzed in duplicate, and the mean values of the tests were given as a result.

### 2.3. Determination of the Activity of Antioxidant Enzymes in Erythrocytes

SOD activity in erythrocytes was determined using the method of Misra and Fridovich [20]. This method evaluates the enzyme’s inhibition of the auto-oxidation reaction of adrenaline to adrenochrome in an alkaline environment (pH 10.2). SOD activity was expressed in U/g Hb. CAT activity was determined using the Beers and Sizer method [21]. The principle of the method is based on lowering the absorbance of the hydrogen peroxide (H_2_O_2_) solution decomposed by the enzyme. CAT activity was expressed in IU/g Hb. GPx activity was determined according to the method of Paglia and Valentine [22]. This method is based on the reaction of hydrogen peroxide decomposition by GPx with simultaneous oxidation of reduced glutathione (GSH), and the results are expressed in U/g Hb.

### 2.4. Determination of TBARS and CD Concentration in Erythrocytes and Blood Plasma

The TBARS level was determined according to the methodology of Buege and Aust [23], modified by Esterbauer and Cheeseman [24]. This method is based on the reaction between lipid peroxidation products and thiobarbituric acid (TBA) in an acidic environment. MDA is the main TBA-reactive product of lipid peroxidation. Therefore, for simplicity, the level of all TBA-reactive substances can be expressed as the concentration of MDA. The concentration of TBARS in erythrocytes was expressed in nmol MDA/g Hb and, in plasma, in nmol MDA/mL of plasma. The concentration of CD was determined according to the method of Sergent et al. [25]. CD are formed in the process of lipid peroxidation as a result of the rearrangement of double bonds after the detachment of a hydrogen atom from the rest of the polyunsaturated fatty acid. They have a characteristic absorbance peak at 233 nm. CD concentration in erythrocytes was expressed in absorbance units per g of Hb (Abs./g Hb). In contrast, plasma concentration of CD was expressed in absorbance units per milliliter of plasma (Abs./mL).

### 2.5. Determination of Vitamin E and A Concentration in Blood Plasma

Vitamin A (retinol) and E (α-tocopherol) concentrations were determined using the HPLC method. The mobile phase was an acetonitrile–methanol solution. Vitamin concentrations were determined using the WorkStation Polaris software and expressed in µg/L.

### 2.6. Determination of the Concentration of 8-iso-PGF2α and 4-Hydroxynonenal in Blood Serum

To determine the concentration of 8-iso-PGF2α and 4-HNE, ready-made analytical kits based on the competitive enzyme immunoassay method (ELISA) were used. 8-iso-PGF2α was determined using a kit from Cloud-Clone Corp. (Wuhan, China), while a kit from CUSABIO (Wuhan, China) was used to determine 4-HNE. The determinations were carried out in accordance with the manufacturers’ instructions. The microplates were coated with monoclonal antibodies specific to the determined parameters. The concentration of the determined parameters was determined using the MARS data analysis software and expressed in pg/mL.

### 2.7. Statistical Analysis

Statistical analysis was carried out using the STATISTICA 12 PL package (Kraków, Poland). The obtained results were subjected to statistical analysis using a one-way analysis of variance (ANOVA) test and post-hoc analysis (HSD Tukey’s test). While performing the analysis, the assumptions of the ANOVA test regarding the homogeneity of variance (Levene’s test) and the evaluation of compliance of the analyzed variables with the normal distribution (Kolmogorow–Smirnov test) were considered. The results were presented as the arithmetic mean ± standard deviation (SD). Differences at the significance level of *p* < 0.05 were considered statistically significant.

## 3. Results

### 3.1. The Concentration of Lipid Peroxidation Products Increases in the Blood after Cold-Water Baths, but Regular Winter Swimming Attenuates This Effect

This study showed a statistically significant increase in the concentration of TBARS in erythrocytes as a result of bathing in cold water at BS (Table 3). At BS-30, the concentration of this lipid peroxidation product was about 37% higher (*p* < 0.001) than at BS-0, while at BS-24 it was almost twice as high (*p* < 0.001) than at BS-0. Statistically significant differences were observed when comparing the concentration of TBARS in the erythrocytes at BS and ES (Table 3). The concentration of the examined parameter ES-0 was about 20% lower (*p* < 0.001) than at BS-0. Similarly, at ES-30 and ES-24, the concentration of TBARS in erythrocytes was approximately 44% (*p* < 0.001) and 61% (*p* < 0.001) lower, respectively, than at BS-30 and BS-24.

The study showed a statistically significant increase in the concentration of TBARS in the blood plasma of winter swimmers as a result of exposure to low ambient temperature at BS (Table 3). The concentration of this parameter increased by about 12% (*p* < 0.001) at BS-30 and by about 17% (*p* < 0.001) at BS-24 compared to BS-0. At ES, the changes in TBARS concentrations in the blood plasma were opposite of those at BS. At ES-30, the concentration of TBARS in the blood plasma decreased by about 13% (*p* < 0.01), while at ES-24 it was about 43% (*p* < 0.001) lower than at ES-0 (Table 3). In addition, the blood plasma TBARS concentration at ES-24 was lower (*p* < 0.001) than at ES-30. In comparing the results of the TBARS concentration measurement in plasma at BS-0 and ES-0, no difference was found. However, the concentration of TBARS in the blood plasma at ES-30 was lower by about 28% (*p* < 0.001) than at BS-30. Moreover, this level at ES-24 was about 55% (*p* < 0.001) lower than at BS-24 (Table 3).

The study showed statistically significant changes in the concentration of CD in erythrocytes at BS (Table 3). At BS-30, the concentration of this LPO marker was about twice as high as BS-0 (*p* < 0.001). At BS-24, it was more than three times higher than at BS-0 (*p* < 0.001). A significant increase in the concentration of CD in erythrocytes was also observed in the tests carried out at ES (Table 3). At ES-30, this concentration was about 61% (*p* < 0.001) higher, and at ES-24 it was about 94% (*p* < 0.001) higher than ES-0. No differences were observed in the results when measuring the concentration of CD in erythrocytes at ES and at BS (Table 3).

At BS, statistically significant changes in plasma CD concentrations were observed both at BS-30 and BS-24 (Table 3); the concentration of CD in blood plasma was almost twice (*p* < 0.001) and more than three times (*p* < 0.001) higher after than before swimming, respectively. At ES-30, the CD concentration increased by about 62% (*p* < 0.001) compared to ES-0, and the concentration of this LPO product at ES-24 was lower (*p* < 0.05) than at ES-30 (Table 3). Moreover, at ES in all three blood samples, the concentration of CD in the blood plasma was lower than at the corresponding dates at BS. Plasma CD concentration was lower by approximately 38% (*p* < 0.001) at ES-0 than at BS-0, lower by 48% (*p* < 0.001) at ES-30 than at BS-30, and lower by 74% (*p* < 0.001) at ES-24 compared to BS-24 (Table 3).

In this study, a statistically significant increase in the concentration of 8-iso-PGF2α was observed due to exposure to a low ambient temperature in a study carried out at BS. The concentration of this parameter at BS-30 was about 37% (*p* < 0.001) higher, and at BS-24, it was about 25% (*p* < 0.001) higher, than BS-0 (Table 3). At ES, changes in the concentration of 8-iso-PGF2α in the blood serum were also observed (Table 3). The concentration of 8-iso-PGF2α in the blood serum at BS-30 increased by about 30% (*p* < 0.01) compared to BS-0. Comparing the concentration of the examined parameter at ES and BS, significant differences in the concentration of 8-iso-PGF2α in the blood serum of the subjects were observed. At ES-30, this concentration was about 37% (*p* < 0.001) lower, and at ES-24, it was about 42% (*p* < 0.001) lower, than BS-30 and ES-24, respectively (Table 3). The conducted studies showed no statistically significant changes in the concentration of 4-HNE in the blood serum of study participants as a result of bathing in cold water, either at BS or ES (Table 3). However, statistically significant differences in 4-HNE concentration were found when comparing the results at ES and BS (Table 3). At ES, the concentration of 4-HNE at all three tests, i.e., ES-0, ES-30, and ES-24, was about 19% (*p* < 0.001) lower than at BS (Table 3).

### 3.2. Winter Swimming Stimulates the Erythrocytic Activity of Antioxidant Enzymes, but Has No Effect on the Blood Plasma Concentration of Vitamins A and E

Repeated cold exposure up-regulated most antioxidant defenses, and this led to an attenuation of most indicators of oxidative stress at baseline and the acute pulses in response to cold exposure. At BS, an increase in catalase activity was observed in the erythrocytes as a result of exposure to low ambient temperature (Table 4). The activity of this enzyme increased by about 18% (*p* < 0.05) at BS-30 and by about 30% (*p* < 0.001) at BS-24 compared to BS-0. At ES-30, no statistically significant change in CAT activity was observed, while at ES-24, the activity of this enzyme was about 17% (*p* < 0.05) higher than at ES-0 (Table 4). In the presented study, no statistically significant differences in CAT activity in the erythrocytes were observed between BS and ES (Table 4). Moreover, no statistically significant changes in SOD activity in the erythrocytes were found due to bathing in cold water at BS (Table 4). At ES-30, the SOD activity decreased by approximately 36% (*p* < 0.001) compared to ES-0 (Table 4). At ES-24, the activity of SOD was about 15% (*p* < 0.001) lower than ES-0 and, at the same time, lower (*p* < 0.001) compared to the activity at ES-30. Comparing the activity of SOD in erythrocytes at BS and ES, statistically significant differences were observed (Table 4). At BS-0, SOD activity was approximately 68% (*p* < 0.001) higher than at ES-0. Moreover, the activity of this enzyme at ES-24 was about 42% (*p* < 0.005) higher than at BS-24. On the other hand, there were no changes in GPx activity in the erythrocytes 30 min after the bath (Table 4). However, at BS-24, the activity of this enzyme was higher by about 95% (*p* < 0.001) than at BS-0. At ES, no changes in GPx activity were observed 30 min after winter swimming (Table 4). GPx activity determined in erythrocytes at ES-0 was about two-and-a-half-fold higher than at BS-0. At ES-30 and ES-24, GPx activity in winter swimmers’ erythrocytes was almost three-fold higher (*p* < 0.001) and twice as high than at BS-30 and BS-24, respectively (Table 4).

This study also compared the concentration of vitamins A and E in blood plasma at BS and ES. The study showed no statistically significant changes in the concentration of vitamin A in blood plasma due to bathing in cold water, either at BS or at ES (Table 4). A significant increase in the concentration of vitamin E by about 28% (*p* < 0.05) at BS-30 compared to BS-0 was observed (Table 4). However, there were no statistically significant differences in the concentration of the tested antioxidant vitamins between BS and ES (Table 4).

## 4. Discussion

The impact of cold on the human organism has been studied for many years. However, in the existing literature, relatively few studies have focused on the effect of low temperatures on people who are not subjected to physical effort. There is no scientific evidence confirming the beneficial effects of recreational exposure to low ambient temperatures on the human organism. In this study, an attempt was made to determine whether swimming per se is a stimulus that causes a disturbance of cell homeostasis and whether regular use of this stimulus leads to adaptive changes in the body concerning markers of oxidative stress.

### 4.1. Cold-Water Baths at the Beginning of the Winter Swimming Season Stimulate Lipid Peroxidation Processes

One of the well-known adverse effects of oxidative stress in the human organism is the intensification of LPO [26]. In this study, a statistically significant increase in the concentration of LPO products, including TBARS and CD both in blood plasma and in erythrocytes, as well as 8-iso-PGF2α in the blood serum of subjects exposed to cold, was observed at BS. An increase in the concentration of these LPO products was observed both 30 min and 24 h after the cold-water bath. These changes suggest that exposure to low ambient temperatures causes a disturbance of the oxidant–antioxidant balance toward the intensification of oxidative processes. The impact of short-term exposure to cold on oxidative stress during winter swimming was demonstrated almost 30 years ago by Siems et al. [27], who observed a decrease in uric acid and an increase in the level of the oxidized form of GSH after a cold-water bath. Disturbance of the oxidant–antioxidant balance as a result of swimming should be associated with the fact that this activity is a strong stress stimulus for the organism because of the large surface exposed to cold water [28,29]. As cold stress builds up, the intensity of muscle tremors increases, engaging more and more muscles, accompanied by an increase in the rate of aerobic metabolism [30]. Increased oxygen consumption is associated with increased generation of ROS, which in turn may lead to an oxidant–antioxidant imbalance and increased oxidative stress [31]. However, it is assumed that increased aerobic metabolism and incomplete oxygen reduction in the respiratory chain during the activation of defense mechanisms against cold may play a vital role in this process [31].

The mechanisms of defense against the cold also include peripheral vasoconstriction, which reduces blood flow and heat loss from the organism [32]. The significant sources of increased generation of ROS during exposure to cold may include ischemia and hypoxia caused by spasms of peripheral blood vessels [33]. Ischemia may be associated with an influx of Ca^2+^ ions into cells [34]. This process leads to the activation of Ca^2+^-dependent enzymes, such as proteases and phospholipases, which in turn results in increased generation of ROS and intensification of oxidative damage. It has been proven that the increase in the production of superoxide anion radicals may be related to the intensity of ischemia [33]. In addition, reduced energy stores during ischemia lead to the accumulation of adenine nucleotides, the breakdown of lipid membrane components, and the accumulation of free fatty acids, including arachidonic acid [35]. During reperfusion, the cooled tissues are heated. During warming, arachidonic acid can be metabolized in the lipoxygenase and cyclooxygenase pathways, which can be a source of oxygen radicals [36]. Activated neutrophils can also damage endothelial cells and increase the permeability of the endothelial cell monolayer by producing ROS [37]. As a result of reperfusion, adenine nucleotides are metabolized via the xanthine oxidase pathway [38]. Elevated xanthine oxidase activity may contribute to the formation of oxygen radicals [36].

The various mechanisms described above allow us to link exposure to low ambient temperatures with increased generation of ROS and the associated intensification of the LPO observed in the presented work. The increase in the concentration of 8-iso-PGF2α, a stable and specific free radical LPO product, observed in the presented study seems to be a fascinating result. It is worth noting that this product does not accumulate in the blood, but is rapidly dissipated, as the half-life of this compound in the blood is approximately 16 min [10]. Therefore, the persistence of high concentrations of 8-iso-PGF2α in the subjects’ blood 24 h after a winter swimming session may indicate progressive LPO. Some authors have indicated that the concentration of TBARS does not necessarily translate into the concentration of MDA [10]. It is recognized that the mere measurement of TBARS concentration reflects an increase in LPO due to oxidative stress [39]. However, in this study, to assess the severity of LPO more accurately, a total of 6 LPO-related parameters were determined, including TBARS in erythrocytes and blood plasma, CD in erythrocytes and blood plasma, and 8-iso-PGF2α and 4-HNE in blood serum.

Interestingly, it should be noted that the cold-water bath did not have a statistically significant effect on the concentration of 4-HNE. It has been shown that cells exposed to mild, transient heat or oxidative stress acquire the ability to catabolize 4-HNE more quickly, making them more resistant to its harmful effects and to apoptosis induced by oxidative stress [40]. Similar mechanisms could explain the results of this study. The metabolism of 4-HNE in the human body consists primarily of its conjugation with GSH in a reaction catalyzed by glutathione S-transferases (GSTs) to form the GS–HNE conjugate [41]. The lack of changes in the concentration of 4-HNE under the influence of cold observed in this study may be due to at least partly to efficient activity of GST. This pathway has not been investigated in the presented study, which may indicate the need to continue the experiment, expanding the panel of parameters studied by including the GSH concentration and GST activity. Increased GST expression is an important mechanism to protect cells from oxidative stress-induced apoptosis [42,43].

### 4.2. Regular Winter Swimming Diminishes the Stimulatory Effect of Cold-Water Baths on Lipid Peroxidation Processes

In this study, similar to the beginning of the season, an increase in the concentration of CD in erythrocytes and blood plasma and an increase in the concentration of 8-iso-PGF2α in blood serum was obtained after a cold-water bath at the end of the season. In turn, the concentration of TBARS in erythrocytes and the concentration of 4-HNE did not change significantly. The concentration of TBARS in blood plasma even decreased due to exposure to low ambient temperatures at ES. The observed changes in the concentration of LPO products are not entirely consistent with the results of our previous research, which included a group of experienced winter swimmers and people who had not used winter baths before [44]. Participants in these studies spent 3 min in water at 0 °C; blood samples for testing were collected before entering the water and at 5 and 30 min after the bath. A decrease in the concentration of TBARS in the erythrocytes of experienced winter swimmers 5 min after leaving the water and a general tendency to decrease the concentration of TBARS in the blood after exposure to cold, also in novice winter swimmers, was demonstrated. Changes in the concentration of TBARS were explained by the rapid and effective removal of lipid peroxidation products as a result of the emerging peripheral hyperemia [44].

The results of studies on the effect of cold on the process of LPO are often contradictory. Akhalaya et al. [45] studied the effect of cold water on antioxidant status in an animal model. They showed that a short, 5-min exposure of mice to cold water (13 °C) caused oxidative stress, manifested by an increase in the concentration of TBARS. Similar results were obtained by Geyikli et al. [46], who studied the impact of a 5-min immersion in water at 4 °C on the concentration of MDA. The authors demonstrated that low temperatures increased the concentration of this LPO product in the blood serum and liver of rats. Dede et al. [47] also investigated the effects of cold-water immersion in rats (3 min, 10–12 °C) and observed higher serum MDA levels after immersion. Accordingly, Ivanova et al. [48] showed an increase in the concentration of TBARS in the blood plasma of rats after a 10-min immersion in a 10% NaCl aqueous solution at −5 °C. On the other hand, Park et al. [49] observed no changes in the concentration of MDA in taekwondo players who, after a fight, underwent a 20-min immersion of the lower limbs up to the knees in water at 10 °C. Sutkowy et al. [50] used cold-water immersion as part of recovery after 30-min of exercise on a bicycle ergometer. They indicated that a 5-min immersion in water at 3 °C decreased the concentration of TBARS in the blood plasma and, therefore, probably reduced the severity of LPO. In other studies, Sutkowy et al. [51] also applied cold-water immersion (5 min, 3 °C) after 30-min of exercise on a cycle ergometer. They did not show any changes in the concentration of the tested LPO products (TBARS, MDA, 8-iso-PGF2α, or 4-HNE) compared to the control group subjected to passive regeneration.

Among the products of LPO determined in this study, a statistically significant increase in concentration due to winter baths, both at BS and ES, concerned only CD and 8-iso-PGF2α. It should be assumed that the peak of conjugated diene formation occurs 30–60 min after oxidative damage, after which these compounds are rapidly metabolized and cleared [52]. CD and lipid hydroperoxides are primary products of LPO, whereas TBARS (including MDA) and 4-HNE are secondary products of LPO, appearing later as a result of increased oxidative damage [53]. The lack of changes in TBARS and 4-HNE at ES may indicate that immersion in cold water did not exceed the organism’s repair capacity. It suggests that the LPO cascade was interrupted, resulting in no increase in the concentration of secondary LPO products. Moreover, the disturbance in the form of increased oxidative processes was only a temporary change.

### 4.3. Winter Swimming Stimulates Selectively the Activity of Antioxidant Enzymes in Erythrocytes, but Has No Effect on the Concentration of Vitamins A and E in the Blood Plasma

In this study, no changes in the activity of SOD were observed after cold-water swimming at BS. However, the lack of changes in the activity of this enzyme does not necessarily indicate a disturbance in the functioning of the antioxidant barrier. Blagojević [54] noticed a decrease in the activity of SOD in organisms adapted to cold. The lack of intensification of the SOD action after cold exposure limits the formation of a highly reactive hydroxyl radical, which seems to be a favorable situation. At ES, a significant decrease in SOD activity was observed 30 min and 24 h after winter swimming. These results are consistent with Geyikli et al. [46], who found that a 5-min immersion in 4 °C water reduced SOD activity in rat erythrocytes.

At BS, an increase in CAT activity was observed. Moreover, GPx activity also increased 24 h after the bath. A similar pattern of changes in the activity of these enzymes was also found at ES. The obtained results may indicate an increase in the concentration of hydrogen peroxide from sources other than SOD activity. Literature data on exposure to low ambient temperature on antioxidant mechanisms are ambiguous. Mila-Kierzenkowska et al. [44], similar to the presented study, did not observe changes in GPx activity 30 min after winter swimming. However, the authors showed a statistically significant increase in CAT activity, indicating the crucial role of this enzyme in neutralizing ROS. Park et al. [49] observed that a 20-min immersion of the lower limbs up to the knees in water at 10 °C increased the activity of SOD and GPx, which confirms its beneficial effect on the activation of antioxidant mechanisms [49]. Akhalaya et al. [45] also observed the activation of antioxidant mechanisms in the form of increased SOD and CAT activity and increased ceruloplasmin concentration in cold-exposed mice. Sutkowy et al. [50], in turn, did not observe changes in the activity of SOD, CAT, and GPx after immersion in cold water (5 min, 3 °C) compared to the control group subjected to passive regeneration at room temperature. Additionally, Sutkowy et al. [51] showed no changes in total antioxidant capacity (TAC) after using the same regeneration regimen as above. Dede et al. [47] observed no changes in SOD and GPx activity in rats after a 3-min immersion in water at 10–12 °C. However, they noted a decrease in the concentration of GSH, which could be the result of the intensification of oxidative processes and the consumption of this low-molecular antioxidant.

Activating small molecule antioxidants to scavenge free radicals is a slower process than activating antioxidant enzymes [55]. In this study, no changes in vitamin A concentration were observed, which suggests that it was not used to inactivate free radicals. On the other hand, an increase in vitamin E concentration was observed at BS-30 compared to BS-0. This may be due to the activation of lipid reserves as an energy source after exposure to cold. These changes seem to protect the body against excessive LPO, in which this vitamin plays a key role [13]. In the available literature, an inverse relationship was described between the intensity of the peroxidation process and the concentration of vitamin E in various tissues [52]. Vitamin E is one of the primary antioxidants involved in scavenging peroxide radicals [56]. However, the concentration of this vitamin at ES-30 and ES-24 did not differ from the ES-0 level, suggesting that LPO did not exceed the organism’s antioxidant capacity.

### 4.4. Regular Winter Swimming Improves Oxidant–Antioxidant Balance, Including Weakened Lipid Peroxidation and Increased Activity of SOD and GPx due to Adaptive Changes

One of the main goals of the research conducted as part of this study was to investigate whether regular swimming during one autumn–winter season affects the level of oxidative stress markers. The results seem to prove the development of adaptive changes consisting of reducing the concentration of LPO products and increasing the efficiency of the antioxidant barrier. The study showed that the concentration of all tested products of LPO, except for CD in erythrocytes, was statistically significantly lower at the end of the swimming season than at its beginning. Although at ES, after bathing in cold water, an increase in the concentration of some of the tested LPO markers was observed, it remained significantly lower than at BS. At ES, before entering the cold water, the concentration was lower than at BS for TBARS in erythrocytes, CD in blood plasma, and 8-iso-PGF2α. In turn, 30 min after the cold-water bath, the concentration of all analyzed markers of LPO was statistically significantly lower than at BS. Accordingly, 24 h after leaving the water, the concentration of all LPO markers was lower at ES than BS. After the whole winter swimming season, the LPO inhibitory mechanisms are more active and reduce LPO faster than at BS. The dynamics and magnitude of these changes suggest that regular cold exposure triggers mechanisms capable of quickly and efficiently clearing LPO products after cold-water immersion.

In addition, this study showed higher SOD and GPx activity at ES than at BS, whereas CAT activity remained unaltered. These results indicate a significant increase in antioxidant capacity due to regular swimming during one autumn–winter season. High SOD activity may indicate the organism’s readiness to fight the superoxide anion radical. The lack of differences in CAT activity, with higher GPx activity, may suggest the appearance of hydrogen peroxide at ES in moderately low concentrations. It also could be suggested that glutathione-dependent antioxidant mechanisms are more important for managing the oxidative stress accompanying cold-water immersion. The expression of the PGC-1α gene has been found to be increased following cold adaptation [57,58]. PGC-1α is a potent mitochondrial respiration and biogenesis stimulator. It regulates ROS metabolism and is required to induce many ROS-detoxifying enzymes, including GPx1 and SOD2 [19]. Some studies have indicated that the PGC-1α protein enhances the synthesis of antioxidant enzymes [59]. The expression of PGC-1α is increased by physiological stimuli, such as cold, leading to mitochondrial biogenesis and increased respiration. PGC-1α is crucial in linking stimuli such as cold to an internal metabolic response such as mitochondrial biogenesis via, among others, NRF transcription factors [60]. Simultaneously, PGC-1α protects the organism from oxidative stress by initiating the anti-ROS program that prevents an increase in intracellular ROS levels. PGC-1α can also be induced by ROS and plays a crucial role in the ROS homeostatic cycle [19]. This data suggests that the increase in the activity of antioxidant enzymes observed in this study due to exposure to cold might be the effect of PGC-1α up-regulation.

Moreover, in this study, no differences in the concentration of vitamins A and E were observed after the swimming season, which suggests that regular bathing in cold water does not lead to noticeable changes in the concentration of these vitamins. As mentioned earlier, cold-water baths may potentially intensify oxidative mechanisms, so it is worth ensuring optimal protection against the possible consequences of oxidative stress through appropriate diet modification, considering the proper supply of vitamins A and E of natural origin [61].

Lubkowska et al. [62] studied the effects of an 8-week session of regular immersion in 5 °C water on the activity of antioxidant enzymes and LPO products in rats. The control group in the study consisted of rodents immersed in water at 36 °C. The authors showed that the efficiency of the antioxidant system was higher in females, with higher SOD activity and higher GSH concentration. They also indicated that exposure to low temperatures increased LPO in tissues. The authors emphasized that regular, repeated cold exposure can be a stressor stimulating pro-oxidative processes and may lead to the emergence of adaptive mechanisms protecting against cold-induced damage [62]. In another study, Lubkowska et al. [63] examined the effect of regular winter swimming (2–3 times a week for 5 months) on antioxidant parameters in healthy humans. However, studies at the beginning and the end of the season used whole-body cryotherapy (3 min, −130 °C) to disturb the oxidant–antioxidant balance. At the end of the season, winter swimmers showed less significant change in total antioxidant status following bathing than they did at the beginning of the season. In contrast to the marked increase in SOD and GPx activity before the start of the study, after 5 months of winter swimming, no changes in SOD and GPx activity were observed after using whole-body cryotherapy. After the swimming season, a significant decrease in the concentration of 8-isoprostanes was also observed [63]. Kaushik and Kaur [64] studied the effect of cold on the function of the antioxidant barrier in rats. The animals were subjected to a 3-week exposure to cold at 7–8 °C, resulting in tissue-specific changes in the antioxidant defense system. Chronic exposure to cold seems to impair the functioning of antioxidant mechanisms in rats, as lower SOD, CAT, and GPx activities were observed after the cold session. However, in those studies, an increase in GST activity was observed in all tissues (except the heart), accompanied by a decrease in GSH levels, which may be attributed to an increase in conjugation with LPO products [30].

It is worth mentioning that cold can intensify the process of thermogenesis caused by muscle tremors, thus increasing ROS generation [65]. However, muscle tremors are a response that more often concerns people not acclimatized to low temperatures because, in experienced winter swimmers, non-shivering thermogenesis and vasomotor responses predominate thermoregulation [66], which confirms that winter swimming effectively induces adaptive changes.

Low levels of oxidative stress may positively affect an organism by stimulating the antioxidant response and inducing adaptation changes. It was observed in the presented study that regular winter swimming leads to an increase in the activity of the main antioxidant enzymes. Interestingly, in the experienced winter swimmers with at least two years of winter bathing examined in this study, the antioxidant barrier seems to be weaker at BS, suggesting that adaptation to low temperatures is not permanent. This may suggest the need to continue the research for more than one autumn–winter season to assess the durability of adaptation changes to cold due to regular winter swimming. The influence of cold on the human body has been studied for many years. However, the vast majority of studies on cold focus on its impact on the effectiveness of post-workout regeneration in the context of reducing the oxidant–antioxidant imbalance [31,59,61,62,63,64,65,66,67,68,69,70,71,72,73]. According to the results of those studies, physical effort of sufficiently high intensity induces metabolic stress and leads to increased generation of ROS in skeletal muscle mitochondria [74]. The influence on human basal metabolism, thermogenesis, and cold tolerance has been reported for thyroid hormones [75]. Thyroxine (T_4_) seems to be a critical substrate for human cold tolerance and habituation to cold, as cold adaptation causes the deiodination of thyroxine (T_4_), thus promoting increased blood triiodothyronine (T_3_) levels [76]. A probable mechanism of adaptation in winter swimmers is an increase in the level of the thyroid hormones triiodothyronine and thyroxine. However, further studies are needed to elucidate changes in thyroid hormone levels in winter swimmers, which we did not measure in this study. The results of the presented study seem to confirm the hypothesis about the beneficial effects of regularly repeated treatments using low ambient temperatures on the human organism. Manolis et al. [29] indicated the potential health benefits of swimming if performed carefully. The hardening mechanism consists of regular and short-term exposure to natural stimuli, such as low temperatures, resulting in an increase in tolerance to their effects [27]. In this study, participants exhibited excellent health conditions, as shown in the body composition analysis reported in Table 2. These factors may positively influence the results. It would be reasonable and interesting to extend the research to other study groups differing, for example, in body composition. It should be considered that for people with abnormal BMI values, e.g., indicating underweight or obesity, adaptive changes might look different.

## 5. Conclusions

In conclusion, regular cold exposure during the winter swimming season causes beneficial changes in oxidant–antioxidant parameters. These changes may translate into improved health and reduced risk of lifestyle diseases. The research results may contribute to the dissemination of winter swimming by demonstrating the beneficial changes caused in the human organism due to this activity. Exposure to low ambient temperatures during a few-minute cold-water bath in the autumn and winter period causes a disturbance of the oxidant–antioxidant balance towards the intensification of oxidation processes, as evidenced by the increase in the concentration of lipid peroxidation indicators in the blood of the subjects. There was no significant contribution of vitamins A and E to antioxidant defense after exposure to cold in experienced winter swimmers, which may suggest that additional supplementation with these vitamins for winter swimmers is not justified. The antioxidant barrier in experienced winter swimmers is strengthened when stimuli stimulate the organism in the form of regular, several-minute exposures to cold. In summary, regular winter swimming does not seem to be an excessive burden for the organism in terms of the intensification of oxidative processes. Moreover, performed once a week in 3- to 5-min sessions, it effectively stimulates the organism to develop adaptive changes. Thus, several-minute sessions of winter swimming once a week can be recommended as an effective method to improve health by inducing positive adaptive changes and strengthening the organism’s antioxidant barrier.

## Figures and Tables

**Table 1:** Eligibility criteria.

Inclusion Criteria	Exclusion Criteria
- Male - Age over 18 - Good general health - At least two years of winter swimming experience - Winter swimming at least once a week throughout the winter swimming season - Willingness to volunteer to participate in the trial and sign the informed consent form	- Active smoking or illicit drug use - Taking any dietary supplements - Chronic diseases

**Table 2:** Characteristics of the study group at baseline: body composition analysis.

Parameter	Mean	±SD
BMI [kg/m^2^] BF [%] FM/FFM [%] MM [%] MM [kg] BM [kg] FM [kg] FFM [kg] TBW [%] TBW [kg]	23.1 16.1 19.2 42.8 35.1 82.1 13.2 68.9 61.7 50.7	1.5 3.9 4.4 3.6 4.1 8.7 4.1 6.7 3.9 4.2

BMI: body mass index; BF: body fat; FFM: fat mass to fat-free mass ratio; MM: muscle mass; BM: body mass; FM: fat mass; FFM: fat-free mass; TBW: total body water.

**Table 3:** The concentration of lipid peroxidation products in the blood of winter swimmers.

Tested Parameter	At the Beginning of the Winter Swimming Season (BS)			At the End of the Winter Swimming Season (ES)		
	BS-0 (Baseline)	BS-30	BS-24	ES-0 (Baseline)	ES-30	ES-24
TBARS concentration in erythrocytes [nmol MDA/g Hb]	30.291 ± 4.3354	41.434 ± 4.2794 ***	58.378 ± 5.8316 ***	23.936 ± 2.7405 ***	23.408 ± 3.5105 ^▲▲▲^	22.549 ± 2.8569 ^○○○^
TBARS concentration in blood plasma [nmol MDA/mL]	0.403 ± 0.0444	0.453 ± 0.0679 ***	0.473 ± 0.0692 ***	0.375 ± 0.0504	0.327 ± 0.0388 ^▲▲▲, □□^	0.212 ± 0.0272 ^○○○, □□□, ■■■^
CD concentration in erythrocytes [Abs./g Hb]	0.017 ± 0.0033	0.034 ± 0.0101 ***	0.052 ± 0.0104 ***	0.018 ± 0.0056	0.029 ± 0.0065 ^□□□^	0.035 ± 0.0141 ^○○○, □□□^
CD concentration in blood plasma [Abs./mL]	0.026 ± 0.0049	0.050 ± 0.0113 ***	0.079 ± 0.0144 ***	0.016 ± 0.0035 ***	0.026 ± 0.0049 ^▲▲▲, □□□^	0.020 ± 0.0056 ^○○○, ■^
8-iso-PGF2α concentration in blood serum [pg/mL]	1248.813 ± 262.4536	1714.269 ± 217.7150 ***	1557.033 ± 243.1226 ***	792.015 ± 264.1342 ***	1027.856 ± 273.3737 ^▲▲▲, □□^	902.232 ± 161.9021 ^○○○^
4-HNE concentration in blood serum [pg/mL]	10.905 ± 1.8538	11.054 ± 1.4700	11.031 ± 1.2811	8.859 ± 1.0329 ***	8.900 ± 1.1346 ^▲▲▲^	8.926 ± 0.9146 ^○○○^

Results are presented as mean value ± standard deviation. TBARS: thiobarbituric acid-reactive substances; CD: conjugated dienes (CD); 8-iso-PGF2α: 8-isoprostaglandin F2α; 4-HNE: 4-hydroxynonenal; BS: beginning winter swimming season; BS-0: before swimming in cold water (baseline) at the beginning winter swimming season; BS-30: 30 min after a bath in cold water at the beginning winter swimming season; BS-24: 24 h after a cold-water bath at the beginning winter swimming season; ES: end of the winter swimming season; ES-0: before swimming in cold water (baseline) at the end of the winter swimming season; ES-30: 30 min after a bath in cold water at the end of the winter swimming season; ES-24: 24 h after a cold-water bath at the end of the winter swimming season; *** *p* < 0.001 vs. BS-0; ^▲▲▲^ *p* < 0.001 vs. BS-30; ^○○○^ *p* < 0.001 vs. BS-24; ^□□^ *p* < 0.01 vs. ES-0; ^□□□^ *p* < 0.001 vs. ES-0; ^■^ *p* < 0.05 vs. ES-30; ^■■■^ *p* < 0.001 vs. ES-30.

**Table 4:** The activity of antioxidant enzymes and the concentration of antioxidant vitamins in the blood plasma of winter swimmers.

Tested Parameter	At the Beginning of the Winter Swimming Season (BS)			At the End of the Winter Swimming Season (ES)		
	BS-0 (Baseline)	BS-30	BS-24	ES-0 (Baseline)	ES-30	ES-24
CAT activity [×10^4^ IU/g Hb]	48.080 ± 8.6771	56.816 ± 11.8414 *	62.576 ± 11.8443 ***	54.837 ± 10.4888	56.663 ± 11.3870	64.322 ± 13.4264 ^□^
SOD activity [U/g Hb]	704.436 ± 92.7566	683.087 ± 57.3460	710.673 ± 45.9504	1185.317 ± 168.0817 ***	752.717 ± 123.7629 ^□□□^	1007.905 ± 206.8254 ^○○○,□□□,■■■^
GPx activity [U/g Hb]	2.995 ± 0.5334	2.735 ± 0.4044	5.850 ± 1.3911 ***	7.290 ± 1.3773 ***	8.236 ± 2.4774 ^▲▲▲^	11.104 ± 2.8143 ^○○○, □□□, ■■■^
Vitamin A concentration [μg/L]	625.610 ± 180.6440	640.546 ± 227.8534	535.498 ± 167.8814	578.380 ± 144.3902	601.680 ± 173.3010	546.483 ± 202.1694
Vitamin E concentration [μg/L]	13.966 ± 5.7379	17.882 ± 6.0835 *	15.128 ± 3.4387	15.082 ± 4.5453	17.788 ± 4.5063	17.664 ± 5.2275

Results are presented as mean value ± standard deviation. CAT: catalase; SOD: superoxide dismutase; GPx: glutathione peroxidase; BS: beginning winter swimming season; BS-0: before swimming in cold water (baseline) at the beginning of winter swimming season; BS-30: 30 min after a bath in cold water at the beginning winter swimming season; BS-24: 24 h after a cold-water bath at the beginning winter swimming season; ES: end of the winter swimming season; ES-0: before swimming in cold water (baseline) at the end of the winter swimming season; ES-30: 30 min after a bath in cold water at the end of the winter swimming season; ES-24: 24 h after a cold water bath at the end of the winter swimming season; * *p* < 0.05 vs. BS-0; *** *p* < 0.001 vs. BS-0; ^▲▲▲^ *p* < 0.001 vs. BS-30; ^○○○^ *p* < 0.001 vs. BS-24; ^□^ *p* < 0.05 vs. ES-0; ^□□□^ *p* < 0.001 vs. ES-0; ^■■■^ *p* < 0.001 vs. ES-30.

## Data Availability

The data presented in this study are available in the main article.
